# Histogram-based models on non-thin section chest CT predict invasiveness of primary lung adenocarcinoma subsolid nodules

**DOI:** 10.1038/s41598-019-42340-5

**Published:** 2019-04-12

**Authors:** Anastasia Oikonomou, Pascal Salazar, Yuchen Zhang, David M. Hwang, Alexander Petersen, Adam A. Dmytriw, Narinder S. Paul, Elsie T. Nguyen

**Affiliations:** 1 Department of Medical Imaging, Sunnybrook Health Sciences Centre, University of Toronto, Toronto, Canada; 2 Vital Images, Minnetonka, USA; 3 Department of Medical Imaging, Toronto General Hospital, University of Toronto, Toronto, Canada; 4 Department of Laboratory Medicine and Molecular Diagnostics, Sunnybrook Health Sciences Centre and Laboratory Medicine Program, Toronto General Hospital, University of Toronto, Toronto, Canada; 5 Department of Statistics, University of California, Santa Barbara, USA; 6 Department of Medical Imaging, London Health Sciences and St Joseph’s Hospital, Western University, London, Canada

## Abstract

109 pathologically proven subsolid nodules (SSN) were segmented by 2 readers on non-thin section chest CT with a lung nodule analysis software followed by extraction of CT attenuation histogram and geometric features. Functional data analysis of histograms provided data driven features (FPC1,2,3) used in further model building. Nodules were classified as pre-invasive (P1, atypical adenomatous hyperplasia and adenocarcinoma *in situ*), minimally invasive (P2) and invasive adenocarcinomas (P3). P1 and P2 were grouped together (T1) versus P3 (T2). Various combinations of features were compared in predictive models for binary nodule classification (T1/T2), using multiple logistic regression and non-linear classifiers. Area under ROC curve (AUC) was used as diagnostic performance criteria. Inter-reader variability was assessed using Cohen’s Kappa and intra-class coefficient (ICC). Three models predicting invasiveness of SSN were selected based on AUC. First model included 87.5 percentile of CT lesion attenuation (Q.875), interquartile range (IQR), volume and maximum/minimum diameter ratio (AUC:0.89, 95%CI:[0.75 1]). Second model included FPC1, volume and diameter ratio (AUC:0.91, 95%CI:[0.77 1]). Third model included FPC1, FPC2 and volume (AUC:0.89, 95%CI:[0.73 1]). Inter-reader variability was excellent (Kappa:0.95, ICC:0.98). Parsimonious models using histogram and geometric features differentiated invasive from minimally invasive/pre-invasive SSN with good predictive performance in non-thin section CT.

## Introduction

Lung cancer is the leading cause of mortality from cancers worldwide^1^. Adenocarcinomas are the most common type of lung cancer^2^. In 2011, the International Association for the Study of Lung Cancer (IASLC), the American Thoracic Society, and the European Respiratory Society introduced a new system of classification for lung adenocarcinomas, separating histological findings into 3 categories: pre-invasive lesions including atypical adenomatous hyperplasia (AAH) and adenocarcinoma *in situ* (AIS), minimally invasive (MIA) and invasive pulmonary adenocarcinoma (IPA)^3^. This classification has significant prognostic and treatment implications for patients since AIS and MIA usually show very slow growth on follow up (FU) CT and a favorable prognosis as opposed to IPA that does not^3^.

On chest CT, lung nodules that appear “ground glass” (GG) or “subsolid” have a higher risk of malignancy than an incidentally detected solid nodule and 75% of subsolid nodules (SSN) are adenocarcinomas^4^. The current Fleischner guidelines recommend that SSN with solid component ≥6 mm or increasing solid component should be considered highly suspicious for lung adenocarcinoma^5–7^. However, about a third of SSN are pre-invasive that could be managed with close FU or completely treated with limited surgical resection, with excellent 5-year survival of up to 100% (curative resection)^8,9^. Furthermore, some patients have multiple SSNs and develop new SSNs during surveillance. Fleischner guidelines provide management recommendations based on the most suspicious appearing nodule(s) based on visual assessment. However, this is a subjective decision and it is also possible that a pure GGN transforms into MIA or IPA over time and pure GGNs may contain an invasive component, which cannot be perceived visually. It is therefore critical to identify noninvasive and objective methods to differentiate SSN representing invasive adenocarcinoma requiring aggressive surgical treatment, from pre-invasive nodules that could remain under surveillance.

Published studies regarding SSN classification were based mostly on thin-slice CT images ≤1.25 mm that may not be routinely used in many institutions mainly due to the large number of images reconstructed, requiring increased time for radiology review and demands on storage capacity for imaging servers. Moreover, lung cancer screening studies may be performed with thicker slice reconstruction (up to 2.5 mm) according to the ACR recommendations^10,11^.

Most SSN will be discovered incidentally on chest CT performed for various clinical indications and the protocol may not routinely include thin high-resolution images through the SSN. This study presents a predictive modeling approach combining individual morphologic and data-driven CT-attenuation features to classify invasiveness of lung adenocarcinoma using routine non-thin section chest CT images reflecting “real world” applicability on routine chest CT.

## Results

**Figure 1:**
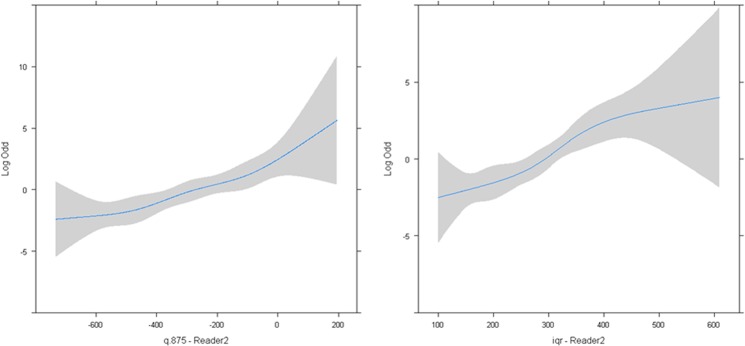
Feature vs. log-odd linearity plot for CT attenuation features: Q.875 (left) and IQR (right) and log odd for the invasive lesion class.

The parameters for CT acquisition techniques, patient characteristics and nodule locations did not show significant differences between the two groups (Table 1). The median and IQR of the geometric and CT attenuation-based predictors are presented in Table 2. The results of the univariate analysis with AUC values, sensitivity, specificity, and optimal threshold (using the Youden index for optimality criteria) are listed in Table 3. CT attenuation features showed higher classification performances with high CT attenuation features such as Q.875 (AUC 0.87), while geometric features such as volume showed lower AUC values (0.74). Multivariate analysis of the main predictors did not reveal significant interaction or non-linearity (all p-values with test >0.05) (Fig. 1).

**Table 1:** CT parameters, patient characteristics and tumor properties of T1 and T2 groups.

Characteristic	T1 (AAH/MIA)	T2 (IPA)
***CT Acquisition***		
CT mA (median & IQR)	50 (87)	50 (110)
CT kV (median & IQR)	120 (15)	120 (15)
***Reconstructed slice thickness (nr nodules)***		
2.5 mm	1	0
3 mm	37	47
5 mm	18	6
***Patient characteristics***		
Gender (male/female)	11/44	14/39
Age, years (median & IQR)	64 (13)	67 (15)
Smoking history (with/without)	47/11	38/18
***Nodule location (nr nodules)***		
LUL	18	15
LLL	9	6
RUL	17	20
RLL	9	8
RML	2	3
Lingula	1	1
***Pathologic subtype (nr nodules)***		
All	56	53
AAH*	3	—
AIS*	24	—
MIA*	29	—
IPA*	—	53

**Table 2:** Geometric and CT attenuation parameters of T1 (AAH/MIA) and T2 (IPA) groups. Median (Inter-Quartile Range).

Parameter (median & IQR)	T1 (AAH/MIA)	T2 (IPA)	Total (*Mann-Whitney test*)
***Geometry***			
Volume, mm^3^	1129 (2218)	3459 (5754)	P < 0.0001
Minimum Diameter, mm	12 (5)	15 (10)	P = 0.0016
Maximum Diameter, mm	16 (7)	25 (14.25)	P < 0.0001
Mean Diameter, mm	14 (6)	20 (14)	P < 0.0001
Max**/**min Diameter Ratio	1.37 (0.40)	1.67 (0.57)	P = 0.0002
Consolidation Ratio	0.29 (0.47)	0.76 (0.43)	P < 0.0001
***CT attenuation - parametric***			
Mean, CT HU	−639 (169)	−442 (225.5)	P < 0.0001
SD CT HU	168 (70)	250 (58)	P < 0.0001
Skewness CT HU	0.56 (0.43)	0.26 (0.68)	P < 0.0001
Kurtosis CT HU	3.22 (1.30)	2.53 (0.80)	P < 0.0001
***CT attenuation - non-parametric***			
Q.50 CT HU	−663 (172)	−462 (286)	P < 0.0001
Q.75 CT HU	−555 (230)	−246 (336)	P < 0.0001
Q.875 CT HU	−463 (236)	−98.25 (279)	P < 0.0001
IQR CT HU	197 (105)	348 (137)	P < 0.0001
***CT attenuation - Functional Principal Components***			
FPC1 CT HU	0.315 (0.433)	−0.246 (0.40)	P < 0.0001
FPC2 CT HU	−0.0012 (0.242)	0.080 (0.291)	P = 0.1473

**Table 3:** Main parameters and ROC-AUC performances - Reader 2.

Parameter	AUC	P-value	Sensitivity	Specificity	Best Threshold
FPC1 CT HU	0.88 [0.80 0.93]	P < 0.0001	77.4%	89.3%	>0.072
SD CT HU	0.88 [0.81 0.94]	P < 0.0001	90.6%	76.8%	>197.85
Q.875 CT HU	0.87 [0.79 0.93]	P < 0.0001	77.4%	87.5%	>−258
IQR CT HU	0.87 [0.79 0.93]	P < 0.0001	75.5%	87.5%	>297
Q.75 CT HU	0.86 [0.78 0.92]	P < 0.0001	81.1%	80.4%	>−401.5
Consolidation Ratio	0.84 [0.76 0.91]	P < 0.0001	69.8%	89.3%	>0.625
Mean CT HU	0.84 [0.76 0.90]	P < 0.0001	81.1%	80.4%	>−543
Q.50 CT HU	0.83 [0.74 0.89]	P < 0.0001	73.6%	80.4%	>−566
Kurtosis CT HU	0.78 [0.69 0.85]	P < 0.0001	67.9%	82.1%	≤2.70
Maximum Diameter	0.76 [0.67 0.84]	P < 0.0001	62.3%	82.1%	>22
Skewness CT HU	0.74 [0.65 0.82]	P < 0.0001	58.5%	85.7%	≤0.306
Volume (log)	0.74 [0.66 0.83]	P < 0.0001	54.7%	85.7%	>3.11
Mean Diameter	0.74 [0.65 0.82]	P < 0.0001	66.0%	78.6%	>17
Diameter ratio	0.71 [0.61 0.79]	P < 0.001	60.4%	73.2%	>1.57
Minimum Diameter	0.68 [0.58 0.76]	P = 0.0008	52.8%	82.1%	>14
FPC2 CT HU	0.58 [0.48 0.67]	P < 0.157	33.96%	89.29%	>0.157

### Functional analysis of the CT attenuation curves

**Figure 2:**
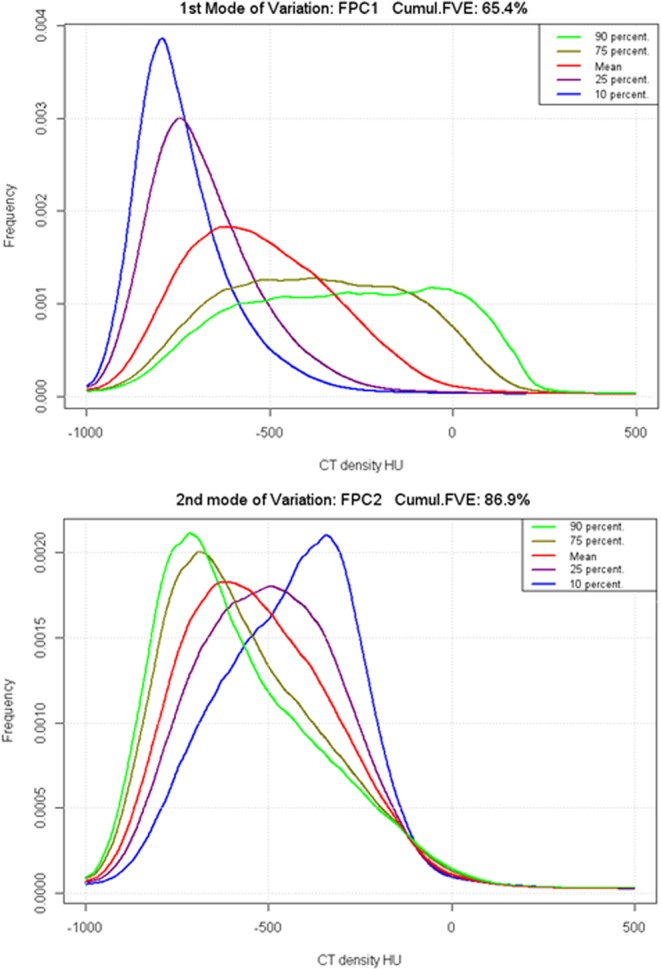
Variation plot for GGO CT density. Left: First mode of variation (FPC1 CTHU). Right: second mode of variation (FPC2 CT HU). Red curves correspond to the mean curves in the nodule data sample.

**Figure 3:**
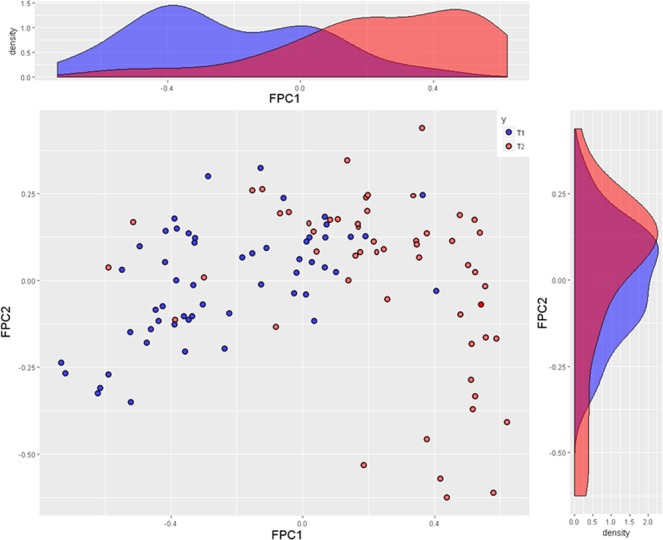
FPC1-FPC2 plot with GGO type and their marginal density distributions.

Separate data driven predictors related to the subsolid nodule CT attenuation histogram were extracted from the histograms using the functional principal component (FPC) analysis. The FPC analysis revealed two main modes of variations among CT attenuation profiles in the dataset (Fig. 2). The FPC1 plot shows a transition from a nodule with mostly low attenuation (10th percentile - blue curve) to more heterogeneous curve profiles (purple, red and brown curves) ending with highly heterogeneous curves including higher attenuations (90th percentile - green curve). The FPC2 plot shows another independent type of variation where a moderately high attenuation profile reversed from low attenuation to high attenuation dominant. This variation of CT attenuation is not associated with heterogeneity change. Both FPCs explain up to 86.9% of the variation for the nodule CT attenuation curves. The lesion CT attenuation curves can be visualized in a 2D scatterplot using only their FPC1 and FPC2 (centered) coordinates, together with their invasiveness class according to pathology; T1 and T2 (Fig. 3). This plot shows a good visual separation of the tumor types based on these two components: higher values of FPC1 are associated with most of the invasive lesions, that is, more heterogenous higher attenuation curves. In contrast, FPC2 presents a less obvious separation pattern of points with invasive lesions. These two FPC components were used in the predictive models for SSN classification.

### Nodule Classification performance based on invasiveness

Three best final linear logistic regression models to predict invasiveness of the SSN were selected based on AUC performances and model parsimony (Table 4). The first model used the two CT attenuation-related predictors - Q.875 and IQR - and two geometric predictors - volume and diameter ratio. The predictive classification performance using the cross-validation gave AUC 0.89 (95%CI:[0.71 1]) and accuracy 81.0% (95%CI:[58.1 94.6]). Mean absolute calibration error was 0.096.

**Table 4:** Predictive performances for subsolid nodule classification.

Model (Multiple Logistic Regression)	AUC [95%CI]	Accuracy [95%CI]	Sensitivity [95%CI]	Specificity [95%CI]
**Model 1: Q**.**875** + **IQR** + **Volume** + **Diameter Ratio**				
Repeated 10-fold CV	0.89 [0.75 1]	81.0% [58.1 94.6]	80.0%	90.9%
**Model 2: FPC1** + **Volume** + **Diameter Ratio**				
Repeated 10-fold CV	0.91 [0.77 1]	81.0% [58.1 94.6]	80.0%	81.8%
**Model 3: FPC1** + **FPC2** + **Volume**				
Repeated 10-fold CV	0.89 [0.73 1]	81.0% [58.1 94.6]	80.0%	81.8%

The second model uses one functional CT attenuation-based predictor - FPC1 - and two geometric predictors - volume and diameter ratio. The cross-validated predictive classification performance was AUC 0.91 (95%CI:[0.77 1]), accuracy: 81% (95%CI:[58.1 94.6]). Mean absolute calibration error was 0.102.

The third model uses two functional CT attenuation-based predictors - FPC1 and FPC2 - and one geometric predictor - volume. In cross-validated classification AUC was 0.89 (95%CI:[0.73 1] and accuracy 81% (95%CI:[58.1 94.6]). Mean absolute calibration error was 0.089.

Models using the following predictors showed comparatively lower classification performances and thus were discarded; minimum diameter, mean diameter, maximum diameter, mean (HU), skewness CT HU, kurtosis CT HU, Q.50 and Q.75, consolidation ratio. Only SD-CT HU could replace IQR in model 1, however no significant performance difference was identified.

Other clinical parameters such as age, gender, upper lobe location and smoking history showed poor performances during the univariate analysis and thus were excluded from the final model. Non-linear classifiers such as SVM did not improve the classification performances and were excluded from the final selection.

### Inter-reader variability of nodule classification performance

The effect of inter-reader variability in the manual correction of lesion segmentation was assessed using model 1 (Table 4). The inter-reader variability was excellent with the Cohen’s Kappa concordance coefficient between each reader predicted class on the testing dataset being 0.95 (95%CI bootstrapped: [0.83 1]) and the intraclass correlation coefficient (ICC) on each reader predicted probabilities being 0.98 (95%CI:[0.96 0.99]).

## Discussion

Conventionally, research on lung nodule classification has been focused on identifying the most efficient imaging biomarkers and optimal cut-off thresholds after linear measurements, visual assessment or lesion segmentation. The ultimate goal is to provide more efficient practical decision-making tools such as lung nodule scoring or reporting systems^12^ to help with risk prediction models for malignancy or degree of invasiveness. In contrast with this approach, machine learning focuses on the highest classification performances, using advanced classifiers with larger feature sets from segmentation^13^. In these models, the interpretation of the individual features is secondary. Results are based on predictive classification performances measured on an independent dataset. Some of these studies based on texture analysis and thin section CT (≤1.25 mm) have shown promising results in differentiating invasive from pre-invasive lesions that present as part-solid or pure GGN^14–16^.

Recently, considerable excitement followed the advances in deep learning and convolutional neural networks (CNN) allowing very high performances on lung nodule classification^17,18^ using the ‘LIDC-IDRI’ lung nodule database (mean reconstructed slice thickness: 1.74 mm)^19^ and smaller private datasets^20^ with AUC performance in lung nodule classification for malignancy well above 0.90. Additionally, CNNs learn features from the data avoiding the burden of manually finding efficient ones in the images and without lesion segmentation. However, deep learning has its own limitations: large datasets of pathology labeled CT cases are needed. Despite active research^17^, the interpretability of the CNN models is very limited giving the user no explanation on the classification result. If no segmentation is needed, current programs usually need a selection of a 3D volume of interest to restrict the computation.

The predictive modeling approach followed in this study integrates known lung nodule geometric features and two types of CT attenuation features: classic a priori features such as median CT attenuation and data-driven features discovered from CT attenuation histograms using an original functional data analysis. This data-driven features discovery reduces the burden of guessing more arbitrary CT attenuation inherent features of the nodule. It also offers a useful insight on the CT attenuation variation inside the lung nodule dataset.

Our results show that models with no more than a couple of CT attenuation features (either quantile-based or FPC) and geometric features (volume and diameter ratio) can differentiate invasive from minimally invasive/pre-invasive SSN with good predictive performance (AUC: 0.89–0.91, accuracy: 81%) and acceptable performances on predicted probabilities (mean absolute error <10%) even in non-thin section images.

**Figure 4:**
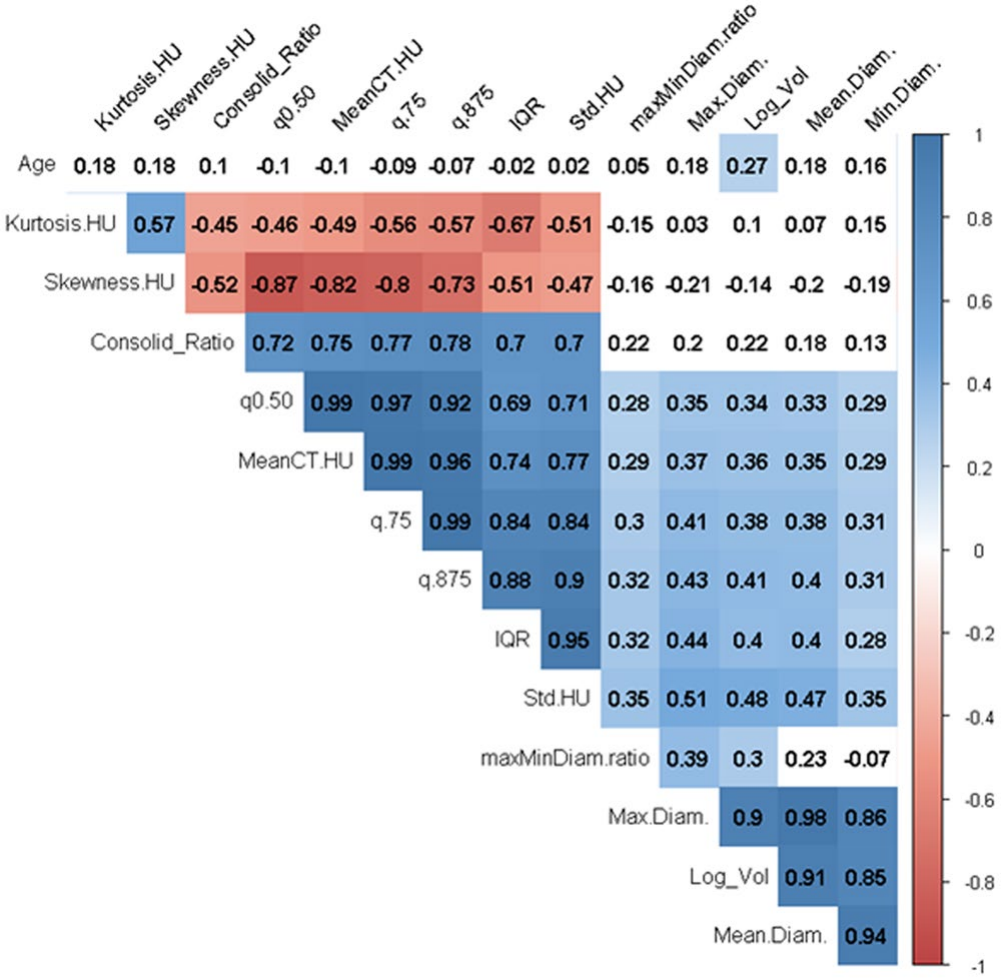
Correlogram for main features. Colored cells correspond to significant correlation test (test for Pearson**’**s correlation based on Fisher**’**s Z transform).

The best CT attenuation features in our multiple logistic models were the 87.5^th^ percentile, the interquartile range (25–75%) and FPC1, while the best geometric feature was the nodule volume. This result is consistent with the previous studies showing the role of nodule heterogeneity (expressed either in texture-related features or in CT attenuation histogram features) and nodule high CT attenuation for SSN classification using thin-slice CT (0.625–1.0 mm). Li Q *et al*. reported AUC 0.824 for 100th percentile combined with maximum diameter in a logistic regression model for discriminating pre-invasive from invasive lesions in pure GGN^21^. Using 1.25 mm slice CT, Ikeda *et al*. found 75^th^ percentile to better differentiate AAH from BAC (AUC: 0.852) (currently classified as AIS). In the same study, the mean CT attenuation outperformed the 75^th^ percentile in discriminating adenocarcinoma from BAC and AAH (AUC 0.871 versus 0.81)^22^. Son *et al*. found that 75^th^ percentile measurement associated with the entropy measurements can help to differentiate invasive adenocarcinoma from pre-invasive lesions (AIS or MIA) with AUC 0.78 (0.71–0.85)^23^. Yagi T *et al*. reported that the 90^th^ percentile together with entropy were independent differentiators of PIA from AIS-MIA with an area under the curve 0.90^24^. Using radiomics with 57 morphologic and texture-based features and Support Vector Machines (SVM), Li M *et al*. found GGN classification accuracy of 0.88 with thin slice datasets (1.25 mm)^11^. 87.5^th^ percentile and 75^th^ percentile are both related to the high CT attenuation and showed a very high correlation (0.99) in our analysis (Fig. 4). IQR on CT attenuation can be interpreted as reflecting the nodule tissue heterogeneity. The FPC1 quantifying the first mode of variation of the CT attenuation curves combined with volume and diameter ratio gives the most accurate model with predictive AUC 0.91. This performance level is comparable to Dey *et al*. results in ‘*MoDenseNet’* deep neural network (AUC 0.90) using dataset of similar size (147 nodules)^20^.

High FPC1 values are simultaneously associated with higher attenuation and higher nodule heterogeneity, while high FPC2 values are mostly linked to increased CT attenuation. These findings are consistent with Son’s *et al*. results using alternative texture-based features of nodule increased heterogeneity, namely increased entropy and reduced uniformity, to differentiate AIS and MIA from IPA^23^. FPCs on CT attenuation histograms are attractive alternatives to the a priori attenuation-based features because of their performances in predictive models and because they can be automatically extracted from CT attenuation histograms with minimal prior knowledge (such as curve smoothness). They also allow exploratory analysis of the modes of variation of the CT density curves in the datasets (Fig. 2). A similar functional data analysis has previously been proposed to classify hyperplastic from adenomatous colon polyps based on optical near-infrared spectra acquired on colonic biopsies^25^.

The consolidation ratio AUC was the lowest (0.84) among the density related predictors in the univariate analysis and slightly reduced the predictive accuracy when combined to other predictors. Therefore, other density related predictors such as FPC1 or IQR were used in the final models.

The nodule volume and diameter ratio are the best geometric predictors of lesion invasiveness when combined in our final models even though their univariate performance (AUC 0.74 and 0.71 respectively) is slightly lower than the best geometric feature, namely the maximum diameter (AUC 0.76). This finding was in agreement with other studies that identified tumor size as independent predictor of invasiveness. Chae HD *et al*. reported that larger mass and lower kurtosis was an independent predictor of invasiveness in subsolid nodules and in their study, mass performed better than volume and diameter^14^. Hwang *et al*. found good classification performances (AUC 0.96) in pure GGN > 5 mm using logistic regression with both nodule mass and texture-related features (entropy, homogeneity), although on a small dataset with only 11 IPA^15^. Eguchi T *et al*. found that the combination of increased tumor size and increased CT attenuation could predict invasiveness in pure GGOs^26^. In another study, maximum diameter and 100^th^ percentile were independent predictors of invasiveness in pure GGOs^21^. In our study, volume and ratio of maximum to minimum diameter were included in the final predictive models for differentiating PIA from MIA-AIS-AAH. The ratio of maximum to minimum diameter reflects more the unique morphology and geometry each tumor as opposed to one dimension only. Another study showed that apart from volume and diameter, the irregular border of a GGO nodule as opposed to round or oval shape is an independent predictor of invasiveness^27^. The small number of predictors, the good linearity between predictors and the logit-transformed probability (log odds) of the tumor invasiveness (Fig. 2), the lack of significant interaction between the main features and the inherent noise due to the dataset variability (various kVP, slice thicknesses, noise levels) make the multiple linear logistic regression competitive compared to less interpretable classifiers such as SVM, which in our study did not demonstrate favorable results.

The inter-reader agreement was excellent. This confirms the high level of reproducibility of the technique used, which was also enabled by the automated segmentation of the nodules and the limited need for manual correction.

This study presents several limitations. First, the retrospective study design may have introduced a selection bias because we only included study subjects who underwent surgical resection because nodule histopathology was used as the reference standard. Second, CT studies were obtained with two different CT scanners and slightly different scanning protocols, which may have affected the CT attenuation. However, the majority of the studies were performed with slice thickness comparable to the American College of Radiology (ACR) LDCT protocol recommendations for lung cancer screening and the comparison of patient characteristics and acquisition parameters did not reveal significant differences between SSN classes^10,11^. The overall number of nodules is relatively small but comparable and larger than many other studies^14–16,21,22,24,26,27^. Predictive classification performance was thus established in a limited number of cases for both training and testing. Nevertheless, our reported predictive performances using cross-validation can be considered as safe estimates even though larger-scale prospective cohort studies on SSN classification are needed to validate these results. Finally, our feature set did not include texture-based metrics such as entropy despite positive results in previously published lung nodule classification studies. The large variability of acquisition, the voxel anisotropy and different slice thicknesses are a known challenge for texture-based features^28^. More importantly, automated texture extraction may not be as available as a CT histogram in clinical practice.

In conclusion, the good performance achieved by the proposed classifying technique could provide radiologists a second-read option for reliably assessing the aggressiveness of SSN and to improve interobserver agreement. Predicting the malignancy risk could also provide the surgeon with confidence in choosing the optimal therapeutic option with closer CT surveillance or sublobar resection reserved with those with more concerning features^13^. Predictive models could be enhanced in the future by integrating proteomics, epigenetic and genetic markers available in non-invasive tests^29,30^.

## Materials and Methods

### Subjects

The study was approved by the research ethics board of a single institution institution and patient consent was waived due to the retrospective nature of the study. All methods were performed in accordance with the relevant guidelines and regulations. SSN with adenocarcinoma diagnosis were identified according to the two following methods: first a search of the pathology database containing all lung resections performed at our institution from January 2013 to August 2016 took place. Secondly, patients were identified from the electronic medical record and radiology information system of our institution using the following search terms: “adenocarcinoma”, “subsolid”, “part-solid” and “GG” under the search for “CT chest procedure” from January 2013 to September 2017. Patients were included in the study if there had been a biopsy or resection demonstrating adenocarcinoma spectrum disease lesions and the pathology was recorded. Patients were excluded if non-contrast CTs were unavailable or if there were multiple nodules of different pathology in the same lobe creating uncertainty during radiology-pathology correlation. An experienced subspecialty pulmonary pathologist (15 years of experience in pulmonary pathology) reviewed the pathologic specimens and confirmed the pathology diagnoses according to the new adenocarcinoma classification. Cases were recruited in chronological order until there were more than 109 cases for pre-invasive, minimally invasive and invasive lesions. Pre-invasive lesions included AAH and AIS according to the new classification^3^. Minimally invasive adenocarcinoma was defined as a small solitary adenocarcinoma (≤3 cm) with a predominantly lepidic pattern and ≤5 mm invasion in greatest dimension in any one focus. IPAs were defined as containing an invasive component >5 mm including subtypes such as lepidic, acinar, papillary, solid, or micropapillary predominant adenocarcinoma^3^.

Clinical and demographic characteristics of the patients including age, sex, smoking history and lung location were recorded from the electronic patient data (Table 1). The study population consisted of 93 patients with 109 SSN pathologically proven and classified as AAH, AIS, MIA and IPA^3^. The nodules were categorized as pre-invasive (P1), minimally invasive (P2) and invasive adenocarcinomas (P3). P1 included AAH and AIS (n = 27), P2 included MIA (n = 29) and P3 included invasive adenocarcinomas (n = 53). All nodules were surgically resected.

### Chest CT Image Acquisition

All CT studies were performed without intravenous contrast medium using one of the 2 scanners in a single institution (Aquilion One 64 and 320 detector row CT, Canon Medical Systems, Otawara, Japan). The CT studies were completed using dose modulation with the following technical parameters: 100–135 kVp, 80–120 mAs; 1–3 mm slice reconstruction, gantry rotation time 0.35 seconds and standard field of view 35–40 cm. All images were reconstructed using a standard kernel with a slice thickness of 1–3 mm (Table 1).

The last CT prior to the date of surgery or biopsy was selected for analysis. CT studies were done in supine position and during full inspiration. The median interval period between the CT study and the date of surgery was 73 days (IQR: 45 days). The median time interval between the biopsy and the resection for all nodules was 49 days (IQR: 73 days). All CT images were anonymized and transferred as DICOM images for analysis on a Vitrea workstation (Vital Images, Minnetonka USA).

### CT morphologic analysis

The CT morphologic analysis of the SSN was done in 2 steps: segmentation of the nodules followed by calculation and extraction of the features.

#### GGO Segmentation

**Figure 5:**
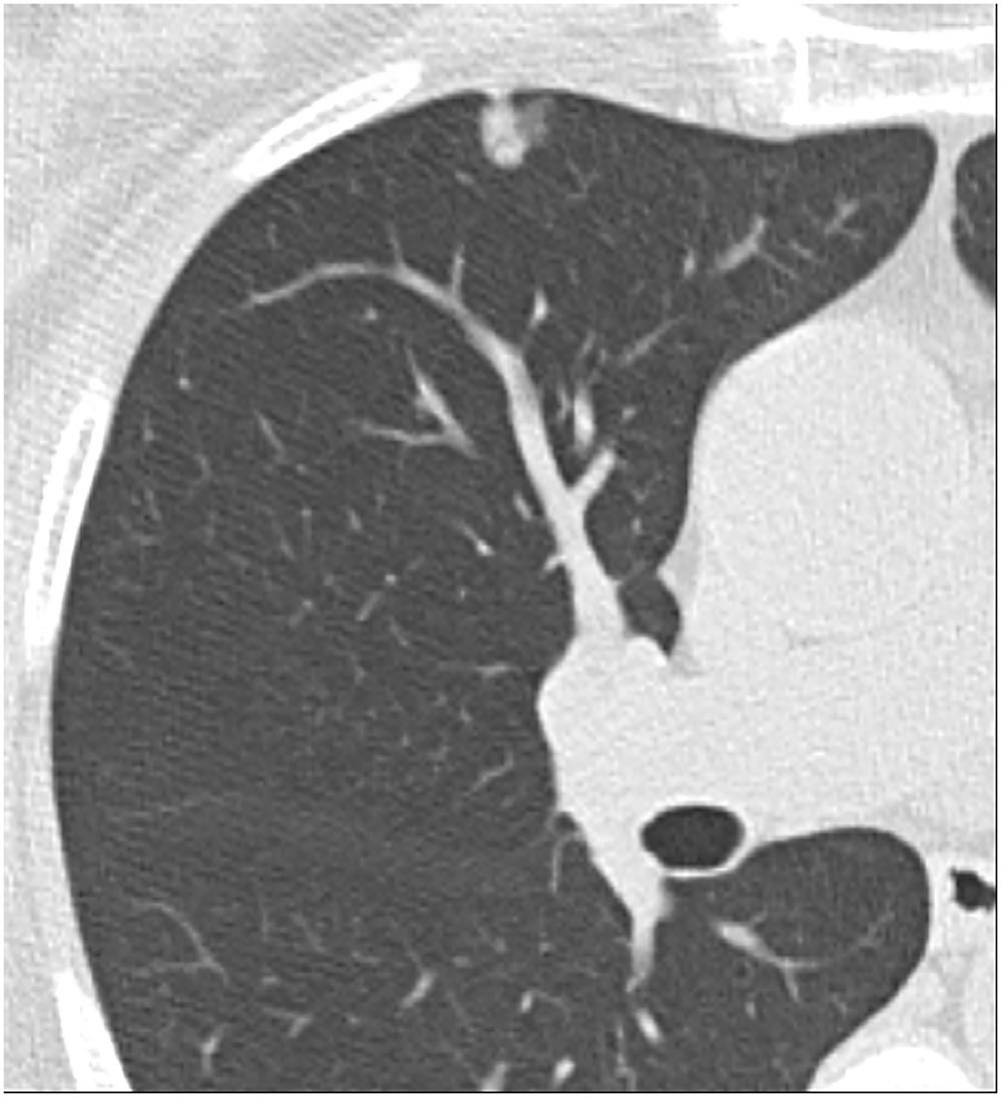
Subsolid nodule with 10 mm solid component and surrounding ground-glass attenuation in a 60-year old non-smoking woman found to have minimally invasive adenocarcinoma at resection.

**Figure 6:**
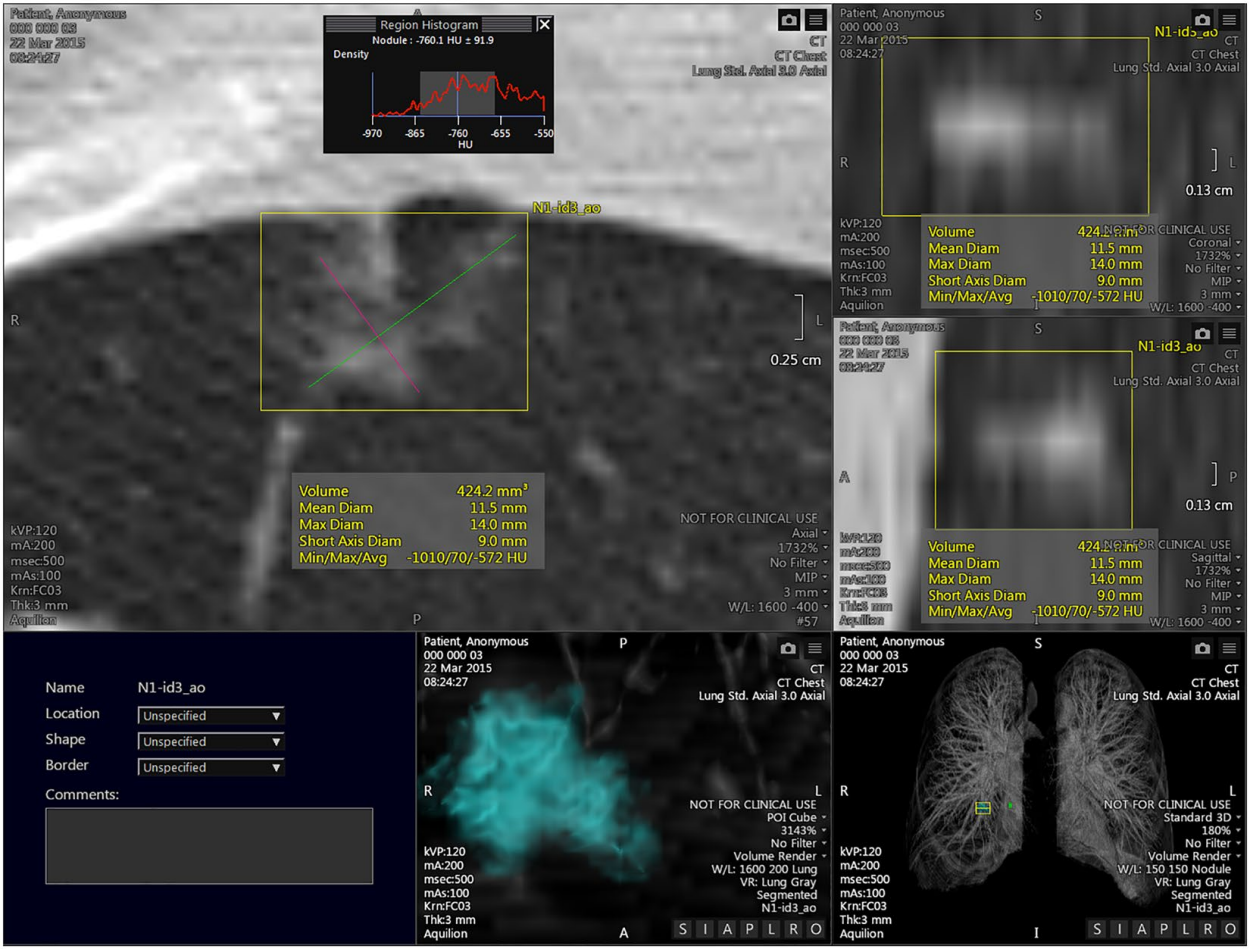
Segmentation analysis of the subsolid nodule demonstrated in Fig. 5, shows the volume and histogram that spans a wide range of attenuation values.

Two chest radiologists with 12 and 16 years of experience respectively, independently evaluated each nodule while blinded to the pathology. The SSNs were segmented using an automated GGN probe of commercial software, Vitrea v7.3. The single-click lung nodule automated segmentation method was applied, and a ROI was automatically drawn after clicking in the centre of the nodule. Manual correction of the contour of the nodules was performed whenever necessary by the 2 independent readers so that the region of interest was delineated around the contour of each nodule as demonstrated by the increased density on each sequential CT slice that the nodule was visible. Vessels were excluded from the region of interest wherever possible (Figs 5, 6).

#### Extraction of imaging features

Analyzed features for each nodule included geometric and CT attenuation parameters. The geometric parameters were automatically generated after nodule segmentation including: (1) volume expressed in log scale, (2) minimum diameter, (3) maximum diameter, (4) mean diameter and (5) lesion irregularity index expressed as the maximum to minimum diameter ratio (Table 2). Each nodule density histogram was automatically created via Vitrea software and exported as.csv file. Further processing was performed using custom tools developed in R statistical programming environment^31^. For each nodule, CT attenuation histogram parametric and nonparametric features were computed. Parametric features included mean, standard deviation, skewness and kurtosis and non-parametric features included quantile-based values for higher lesion CT attenuation; Q.875 (at 87.5 percentile), Q.75 (at 75% percentile), Q.50 median (at 50% percentile) and IQR mean (Inter-Quartile Range) (Table 2). Additionally, consolidation ratio expressed as max consolidation (mm)/max tumor diameter (mm) was computed^32^. These features were preselected for their putative predictive values based on the existing literature on SSN classification. Besides this conventional approach, an alternative data-driven functional analysis of the CT attenuation histograms was performed to extract relevant features directly from the curves without a priori knowledge^25,33–36^. Two functional principal components (FPC) explaining most of the variation in our sample of CT attenuation histograms (Table 2) were added to our list of predictors for subsequent model building.

### Statistical analysis

For the purpose of this study we grouped pre-invasive (P1, AAH/AIS) and minimally invasive (P2, MIA) together as T1 (n = 56) given their similar survival rates and PIA (P3) as T2 (n = 53)^16^. Variable selection was performed on the nodule features from the segmentation using the univariate variable importance based on ROC-AUC performance (Table 3). Highly correlated variable groups were visualized using a correlogram with Pearson correlation and highly correlated variables with lower performances were discarded (Fig. 4).

A functional data analysis was performed on the CT attenuation curves to visualize their main modes of variation in the nodule population and to extract data-driven CT attenuation features for tumor classification. Original CT histograms were converted in smooth curves defined between −1000 HU and 500 HU using Ramsey’s method for frequency distributions^33^. Two FPC explaining 86.9% percent of the variation between CT attenuation curves were selected following Petersen & Müller’s FPC method for frequency distributions using the R-library “fdadensity”^37^. The two resulting FPC (FPC1 and FPC2) are non-correlated new variables related to the variation of the CT attenuation curves presented in the result section.

In a multivariate analysis, the linearity assumptions and the presence of interaction for the main predictors in logistic regression models were tested using non-linear regression plots, ANOVA and Wald tests^38^. Performances of the baseline features in binary nodule classification (T1/T2), were assessed using the following models: multiple (linear) logistic regression and non-linear classifiers including support vector machines (SVM) with radial kernel and polynomial kernel. SVM fast tuning was performed using repeated (100 times) 10-fold cross validation on the training sample using the R-library ‘Caret’ with multicore parallelization^39^. The predictive performance of the classification models was assessed using a repeated 10-fold cross-validation (100 repeats). The area under the ROC curve (AUC) was used as diagnostic performance criteria. Accuracy, sensitivity and specificity were also computed. Besides the evaluation of predictive accuracy, calibration of the classification models was assessed to verify the validity of the predicted probabilities using the bootstrapped calibration curves for logistic models with Harrell’s method^38^.

The 2 groups of datasets that were generated by each reader separately were assessed for inter-reader variability using Cohen’s Kappa coefficient and intra-class coefficient (ICC) on the predicted tumor classes and probabilities. A two-sided p-value less than 0.05 was chosen to indicate a statistically significant difference.

## Data Availability

The datasets generated during and/or analyzed during the current study are available from the corresponding author on reasonable request.
